# Plasma levels of complement proteins from the alternative pathway in patients with age-related macular degeneration are independent of Complement Factor H Tyr^402^His polymorphism

**Published:** 2012-08-30

**Authors:** Aldacilene Souza Silva, Anderson Gustavo Teixeira, Lorena Bavia, Fabio Lin, Roberta Velletri, Rubens Belfort, Lourdes Isaac

**Affiliations:** 1 Department of Immunology, Institute of Biomedical Sciences, University of São Paulo, São Paulo, Brazil; 2 Department of Ophthalmology, Federal University of São Paulo, São Paulo, Brazil

## Abstract

**Purpose:**

To investigate the influence of the Factor H (CFH) Tyr^402^His polymorphism on the plasma levels of the alternative pathway proteins CFH, C3, Factor B (FB), Factor D (FD), and Factor I (FI) and the inflammatory marker C-reactive protein (CRP) in 119 patients with age-related macular degeneration (AMD) and 152 unrelated control individuals.

**Methods:**

Patients with AMD and the control group were separated according to CFH polymorphism, age, and gender. Plasma complement proteins and CRP concentrations were determined with enzyme-linked immunosorbent assay, immunodiffusion, or nephelometry.

**Results:**

Significant differences in the concentrations of FD and FI were observed between the patients with AMD and the control individuals. We observed significantly reduced FD plasma levels in patients with AMD. We also identified a significant decrease in CFH plasma levels in female patients with AMD in relation to female controls. Plasma FI levels were significantly increased in patients with AMD compared to the control group. Regarding gender, a significant increase in FI plasma levels was observed in male patients. Finally, we found no significant correlation between the CFH Tyr^402^His polymorphism and the CFH, C3, FB, FD, FI, and CRP plasma levels.

**Conclusions:**

Patients with AMD present altered levels of FD and FI in a manner independent of this CFH polymorphism, and gender apparently contributes to the plasma levels of these two proteins in patients with AMD and control individuals.

## Introduction

The complement system plays an important role in host defense as a central component of innate and acquired immunity [1,2] and is considered one of the most effective protagonists of the immune and inflammatory responses [3]. An understanding of the complement system’s activation, regulation, and effector mechanisms is important to guide the search for new therapy targets for pathological conditions. During homeostatic conditions, the complement system is strictly regulated by soluble and cell membrane–associated proteins. When deregulated, this system can induce damage to host cells and consequently contribute to the development of specific diseases and pathological conditions such as certain autoimmune diseases, glomerulonephritis [4] and hemolytic uremic syndrome [5].

The complement system is activated as a cascade by the classical, alternative, or lectin pathways. The alternative pathway is continuously activated in vivo through spontaneous C3 thioester bond hydrolysis, resulting in the formation of C3 convertase, generating C3b, and other activated proteins that mediate many biologic functions of the complement system [2,6]. Factor H (CFH) is one of the main regulators of the activation of the alternative pathway. CFH prevents the formation of the C3 convertase enzyme and promotes its dissociation. In addition, CFH acts as a cofactor of the enzyme factor I (FI), mediating the proteolytic inactivation of C3b [7].

The inflammatory response contributes effectively to important diseases related to aging, including Alzheimer disease and Parkinson disease [8], multiple sclerosis [9], and atherosclerosis [10]. Recent studies have provided increasing evidence that inflammation is an important mechanism in age-related macular degeneration (AMD) etiopathogenesis [11-13]. Furthermore, elevated levels of serum C-reactive protein (CRP), a well known inflammation marker, are associated with AMD [14].

AMD is the most common cause of irreversible visual loss in the elderly population of the Western world [15]. This late-onset disorder severely undermines vision, causing progressive destruction of the macula, a noble region of the retina, and a consequent loss of macular function and death of photoreceptors cells [16]. AMD is characterized by the presence of drusen, a focal deposition of extracellular material underneath the retinal pigment epithelium. Despite intensive investigation, many fundamental questions regarding AMD pathogenesis remain unclear.

A breakdown of the blood–retinal barrier is observed in neovascular patients with AMD and is associated with retinal edema and the inflammatory repair process [17]. Under these conditions, complement circulating proteins may reach the retinal region and may locally activate the alternative pathway. Close regulation of this system is essential to avoid continuous amplification of the inflammatory process in the eye. Among the many different substances encountered in the drusens of patients with AMD are the complement proteins C1q, C3, C4, C5, CFH, membrane cofactor protein, decay accelerating factor, the anaphylatoxins C3a and C5a, immunoglobulins, CRP, cholesterol esters, and low-density lipoprotein [18,19]. These results strongly indicate that the complement system is activated in situ at the macula and if unregulated could possibly contribute to inflammatory response in the eye.

Since 2005, numerous genetic studies have suggested that AMD might be associated with the CFH Tyr^402^His polymorphism, since homozygous individuals carrying the variant CFH His^402^ are five to seven times more susceptible to developing AMD [20-22]. Many of these studies explored this association in an attempt to highlight the mechanisms by which CFH can participate in the pathogenesis of AMD. Recently, researchers have shown that CFH is one of the most abundant proteins that bind to malondialdehyde (MDA)—a common product of lipid peroxidation generated after oxidative stress [23]. MDA binds to local tissue proteins and may trigger an inflammatory response as has been observed previously in atherosclerosis [23] and AMD [24,25]. The variant CFH His^402^ binds to MDA proteins less efficiently than CFH Tyr^402^ and so could be a possible explanation for the higher risk of developing AMD observed in individuals carrying the variant CFH His^402^ [23].

Recently, we demonstrated that the CFH Tyr^402^His polymorphism is a risk factor for developing AMD in Brazilian patients: an odds ratio of 1.36 for patients carrying only one 1277C allele (heterozygous CT; His/Tyr) and 4.63 for those carrying two 1277C alleles (homozygous CC; His/His) compared to the control group [26].

Since the retinal region of patients with AMD may be exposed to blood circulating proteins and activation of the complement system could enhance the inflammatory process, we decided to investigate if patients with AMD with different CFH variants (Tyr^402^His) present differences in the plasma levels of the complement alternative pathway proteins CFH, C3, FB, FD, and FI. Because AMD is an inflammatory disease, we also characterized the inflammatory status of these patients by determining their serum CRP levels.

## Materials

### Human participants and plasma samples

Blood was withdrawn using 0.34 M EDTA and after 30 min at room temperature the samples were centrifuged and plasma harvested and aliquot and kept at −80 °C until use. Blood samples were obtained from a total of 119 patients with AMD and 152 unrelated controls previously described in a prospective investigation in which they were genotyped for CFH Tyr^402^His polymorphism [26]. All participants were over 50 years of age, underwent a complete ophthalmoscopy examination, and provided informed consent for inclusion in the study and the use of blood and DNA samples for scientific purposes. The control subjects were examined with a dilated fundus and selected if no signs of AMD or other retinal disorders were detected. The study was approved by the Ethics Committee for Human Research of the Institute of Biomedical Sciences of the University of São Paulo, São Paulo, Brazil.

Baseline characteristics of the investigated group population have been described previously [26]. Mean ages of the patients with AMD and controls were 73.3±9.1 and 71.7±9.7, respectively. Gender distribution was 39% (46/119) male and 61% (73/119) female in the AMD group and 34% (51/152) male and 66% (101/152) female in the control group. No statistical difference was observed between patients with AMD and controls with respect to age or gender. The distribution of CFH phenotypes among patients with AMD was significantly different from that among the control subjects (χ^2^=22.025, p<0.001) [26].

### Plasma levels of complement proteins and C-reactive protein

Plasma levels of C3, FD, and FI were determined with enzyme-linked immunosorbent assay (ELISA). Microtiter plates (Costar, #3590, Corning, New York, NY) were coated with a capture specific antibody for each protein, using polyclonal rabbit anti-human C3, rabbit anti-human FD, or monoclonal mouse anti-human FI as the capture antibody (Calbiochem, Darmstadt, Germany). Goat anti-human C3, anti-human FD, or anti-human FI (Calbiochem) were used as secondary antibodies. Detection was performed using an alkaline-phosphatase conjugated antibody anti-goat immunoglobulin G (IgG) and p-nitrophenyl phosphate (pNPP; Calbiochem) substrate. Optical density was measured at 405 nm. The assay was calibrated with different concentrations of purified C3, FD, or FI proteins (Calbiochem). Plasma levels of FH and FB were determined using a radial immunodiffusion protocol [27]. Plasma levels of CRP were determined with nephelometry.

### Statistical analysis

In all experiments, the AMD group was compared to the control group. The categorical data between the two groups were analyzed, and the Hardy–Weinberg equilibrium was tested using the χ^2^ test. Numerical data were examined using the Mann–Whitney test and ANOVA (ANOVA) analysis. The significance level was set at p<0.05. All data were expressed as the mean and standard deviation.

## Results

**Figure 1:**
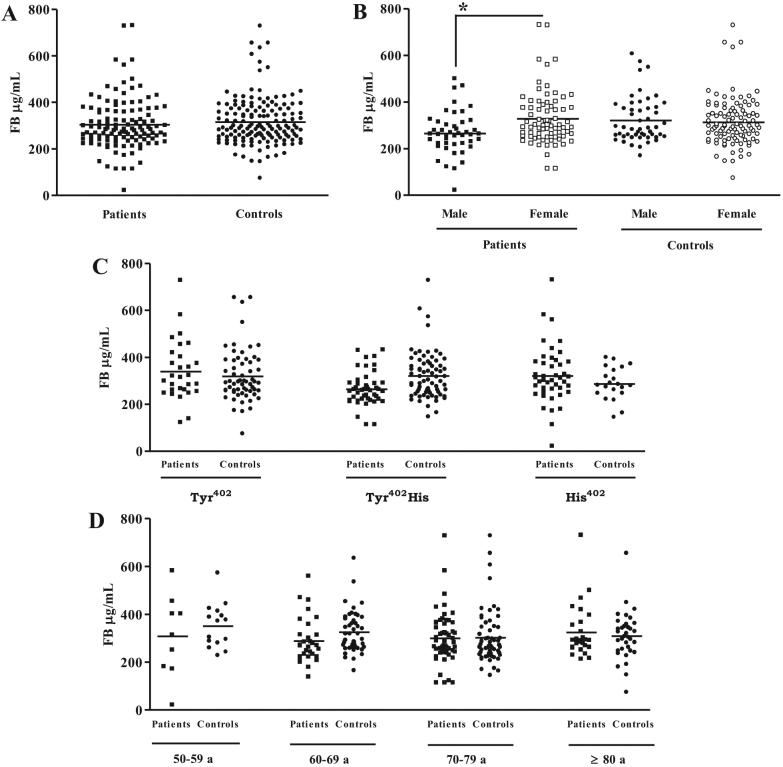
Plasma factor B levels. The plasma factor B (FB) concentration in samples from 119 patients with age-related macular degeneration (AMD) and 152 controls was determined with enzyme-linked immunosorbent assay (ELISA; **A**). The AMD patient group was made up of 46 men and 73 women. In the control group, 51 were male and 101 female (**B**). FB levels are also classified according to factor H (FH) polymorphism (**C**) and age (**D**). *p<0.05.

We assessed whether the CFH Tyr^402^His polymorphism was correlated with CFH plasma levels in patients with AMD and control groups. The average concentration of this regulatory protein was similar between the two groups (653.2±194.3 µg/ml for patients with AMD; 628.6±182.2 µg/ml for the control group). No significant differences in serum CFH levels were observed in the different age groups (data not shown) or in the groups with different CFH phenotypes (Table 1). We observed a small but statistically insignificant difference between the AMD and control groups in the gender distribution according the levels of plasma CFH: men presented lower levels (612.9±164.4 µg/ml) than women (678.5±208.2 µg/ml) in the AMD group while in the control group, the opposite relationship was observed (men: 645.9±196.8 µg/ml; women: 619.9±174.8 µg/ml; Table 1). Similar results were observed for plasma C3 levels. No differences were found between the two main groups: 1.5±0.7 mg/ml for patients with AMD and 1.6±1.0 mg/ml for the control group. No significant differences were observed when we compared C3 levels accordingly to CFH (Tyr^402^His) phenotype, gender (Table 1), and age (data not shown). We extended this analysis to other complement proteins involved in the alternative pathway. No difference was observed in the FB plasma levels of patients with AMD (303.5±111.0 µg/ml) and the control group (315.4±101.9 µg/ml; Figure 1A). However, we observed a significant increase in the FB plasma levels in female patients with AMD compared to male patients with AMD (327.7±115.9 µg/ml for women; 265.0±91.5 µg/ml for men, Figure 1B). No significant differences in FB plasma levels were observed when the individuals were classified by either CFH (Tyr^402^His) phenotype (Figure 1C) or age (Figure 1D).

**Table 1:** Plasma levels of FH, C3 and CRP in AMD patients and controls according to FH phenotype and gender.

	**FH Phenotype**						**Gender**			
	**Y^402^**		**Y^402^H**		**H^402^**		**AMD**		**Control**	
**Protein (mg/ml)**	**AMD**	**Control**	**AMD**	**Control**	**AMD**	**Control**	**Male**	**Female**	**Male**	**Female**
CFH	0.7±0.2	0.6±0.1	0.7±0.1	0.6±0.2	0.7±0.2	0.6±0.1	0.6±0.2	0.7±0.2	0.6±0.2	0.6±0.2
C3	1.6±0.6	1.5±1	1.5±0.7	1.7±1.1	1.6±0.8	1.5±0.8	1.3±0.7	1.7±0.7	1.5±0.9	1.6±1.1
CRP	0.9±3.7	0.4±0.4	0.5±0.5	0.4±0.5	0.7±1.5	0.6±1.1	1± 3.2	0.5±0.5	0.4±0.5	0.5±0.6

**Figure 2:**
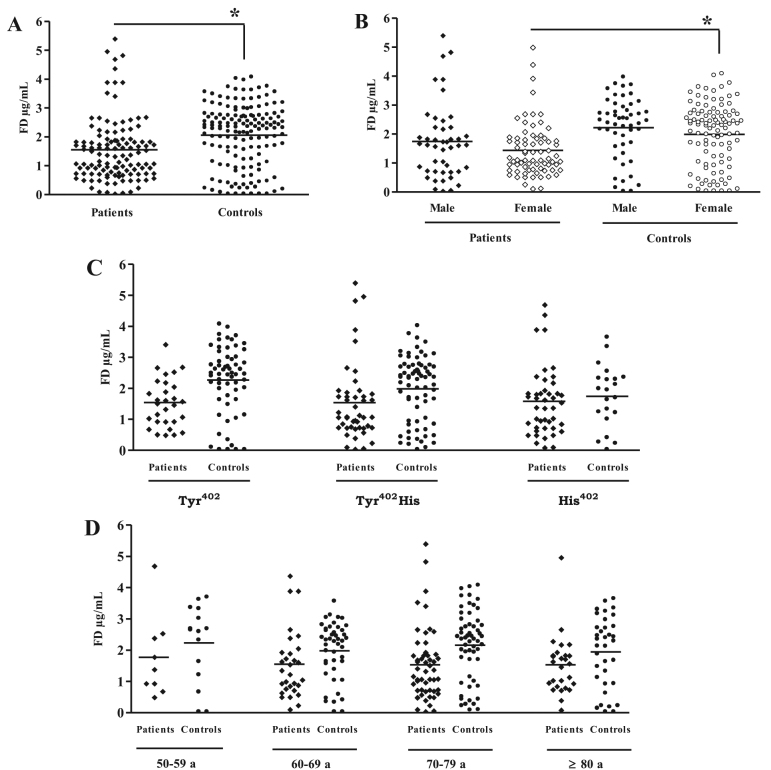
Plasma factor D levels. The plasma factor D (FD) concentration was determined with enzyme-linked immunosorbent assay (ELISA) in samples from 119 patients with age-related macular degeneration (AMD) and 152 controls (**A**). The AMD patient group was made up of 46 men and 73 women. In the control group, 51 were male and 101 female (**B**). FD levels are also classified according to factor H (FH) polymorphism (**C**) and age (**D**). *p<0.05.

Plasma FD levels were significantly decreased in patients with AMD (1.6±1.1 µg/ml for patients; 2.1±1.1 µg/ml, for controls) as shown in Figure 2A. In addition, we found a significant difference in FD levels between female patients and female controls while we did not observe a significant difference in FD levels between the male AMD and control groups (Figure 2B). No significant differences were observed when the individuals were classified by either CFH (Tyr^402^His) phenotype (Figure 2C) or age (Figure 2D).

**Figure 3:**
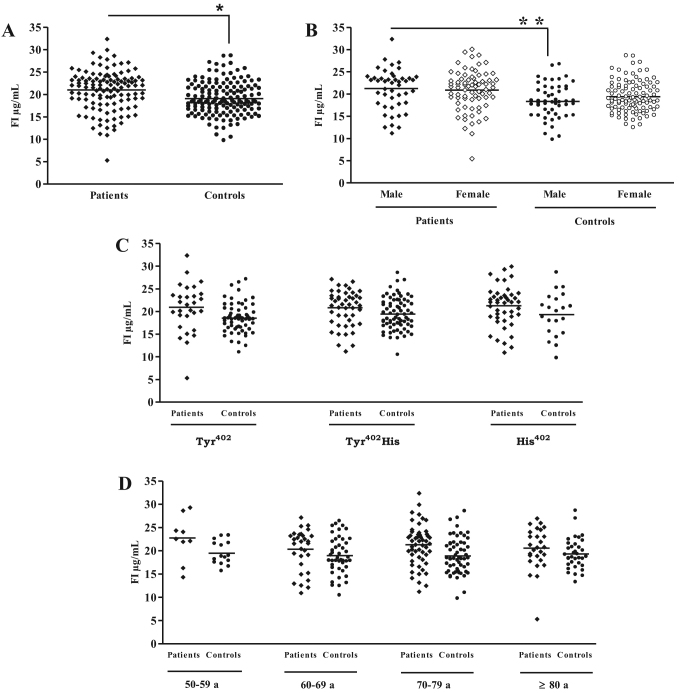
Plasma factor I levels. The plasma factor I (FI) concentration was determined with enzyme-linked immunosorbent assay (ELISA) in samples from 119 patients with age-related macular degeneration (AMD) and 152 controls (**A**). The AMD patient group was made up of 46 men and 73 women. In the control group, 51 were male and 101 female (**B**). FI levels are also classified according to factor H (FH) polymorphism (**C**) and age (**D**). *p<0.05.

FI levels, on the other hand, were observed to be significantly elevated in patients with AMD (21.0±4.5 µg/ml) compared to controls (19.0±3.7 µg/ml, Figure 3A). When we analyzed the FI levels according to gender, we observed a significant difference between male patients with AMD and male controls while observing no significant difference in FI levels between the female AMD and female control groups (Figure 3B). No significant differences were observed when the individuals were classified by either CFH (Tyr^402^His) phenotype (Figure 3C) or age (Figure 3D). These data suggest that gender may exert some influence on FD and FI plasma levels in patients with AMD.

To investigate the association of the CFH Tyr^402^His polymorphism with the systemic inflammatory response, we determined the CRP levels of patients with AMD and control subjects. In our study, most of the patients with AMD presented normal levels of CRP. The CRP concentrations were not statistically different between the patients' and the controls' plasma levels. This parameter was also not influenced by age, CFH Tyr^402^His phenotype, or gender (data not shown).

Age does not seem to influence the concentrations of the alternative pathway proteins that were analyzed. However, for every age category, patients with AMD tend to have higher FI and CRP levels than the control individuals. However, no significant difference was found between these groups in each age category. Furthermore, we found no significant influence of the CFH Tyr^402^His polymorphism on the plasma concentrations of the alternative pathway proteins tested (Table 1).

## Discussion

The etiopathogenic factors of AMD have been studied considerably in recent decades. Currently, AMD is defined as a group of progressive degenerative changes that affect patients over 50 years of age. AMD is clinically characterized by the presence of drusens in the macula [28,29], a crucial area of the retina responsible for central vision.

The correlation between the CFH Tyr^402^His polymorphism and AMD has previously been evaluated by several groups [20-22]. An extensive study employing 4,484 patients with AMD and 5,736 controls from different populations was recently published by Sivakumaran et al. [29]. They suggested that a 32 kb region in CFH downstream of rs1061170 (encoding His^402^Tyr) carries two important single nucleotide polymorphisms with an even stronger association with AMD risk than rs1061170: rs139428 and rs203687 located in intronic regions of CFH.

We examined the CFH Tyr^402^His polymorphism in Brazilian patients [30] and found odds ratios of 1.36 and 4.63 for patients with AMD carrying one or two alleles (1277C; His^402^), respectively, compared to the control group. Our results were similar to those observed in several other populations from North America [20-22,31-33], Europe [34-37], Asia [38-40], and Israel [41]. Due to the ethnically heterogeneous characteristics of the Brazilian population, identifying a genetic correlation with a disease is of great importance.

Several groups have indicated that inflammatory processes play an important role in the pathogenesis of AMD [13]. As an important mediator of inflammation, the complement system is potentially a major player in AMD pathology, an idea supported by the association of polymorphisms of CFH with this disease. Moreover, CFH and other complement proteins, CRP, and cholesterol have been detected in the drusen isolated from the macular region, the sub-retinal pigment epithelium space, and around the capillaries of the choroids [20-22,42]. These observations reinforce the hypothesis that local inflammation along with altered regulation of the complement system on the retina may contribute to the development of AMD [42,43]. Other risk factors for AMD, including smoking, hypertension, and obesity, have been associated with reduced serum concentrations of CFH [44-46], and subtle variations in plasma components of the alternative pathway could have a significant impact on its local activation in response to stimuli even though the researchers found no differences between the AMD and control groups regarding the CFH and C3 levels. The results agree with the results of the present study (Table 1). Even though the FD levels decreased and the FI levels increased in the serum of patients with AMD compared to the control group, our results indicate that the CFH Tyr^402^His polymorphism does not interfere in the plasma concentrations of the components of the alternative pathway of complement activation.

Other studies have shown that the FB Arg^32^Gln polymorphism is associated with a lower risk of developing AMD [47-49]. Expression of FB increases with advancing age in the apical region of the retinal pigment epithelium [50]. In addition, FB levels may be influenced by the action of tumor necrosis factor α (TNF-α) and interferon α (IFN-α). An inverse relationship between FB and AMD independent of genotype was observed by Reynolds et al. [51]. When they assessed the interaction between genotype and complement components, the researchers concluded that this inverse relationship was valid only with the protective FB genotype CC, but was unrelated to AMD risk with the other FB genotypes (CT and TT). Little is known about the concentration of FB in patients with AMD. High levels of FB in patients with AMD compared to controls (mean 803 µg/ml for patients, ranging from 497 µg/ml to 1489 µg/ml; controls with an average of 642 µg/ml, ranging from 378 µg/ml to 1354 µg/ml) have been reported [47]. In our study group, however, we found no significant difference in the plasma concentration of FB between the groups. Moreover, the FB levels observed by Scholl et al. [47] were much higher than those found in our study population; our patients presented an average FB concentration of 303±111 µg/ml while the control subjects had an average of 315±102 µg/ml.

Our observations regarding FD levels were also different from those observed by Scholl et al. [47]: patients: 1.26 µg/ml (0.69–2.30; this study: patients: 1.6±1.1 µg/ml), controls: 0.95 µg/ml (0.50–1.65; this study: 2.0±1.1 µg/ml). More recently, Stanton et al. [52] also observed that the FD plasma concentration was increased in patients with AMD compared to the control group. They also reported a genetic association of a single nucleotide polymorphism rs3826945 and AMD, especially in female patients. FD is a single polypeptide chain of 25 kDa that plays an important role in amplifying the alternative pathway. This heat-labile enzyme is present in the serum in active form [53], and the enzyme’s function is to cleave FB, forming Ba and Bb. FD has an essential role in the onset and amplification of the alternative pathway. FD-deficient mice have a higher vulnerability to retinal damage induced by exposure to sunlight [54]. In addition, patients with total or partial FD deficiency are rare and do not necessarily present higher susceptibility to infection by several microorganisms [55-57]. Recently, another group [58] reported the lack of association between six *FD* single nucleotide polymorphisms and genetic susceptibility to developing advanced AMD. Considering these data along with our results, it seems plausible that early development of AMD may be triggered by an infectious process in susceptible individuals due to imbalances in development and/or regulation of the inflammatory response in which the complement system has a major role [59-61]. However, subtle alterations in activation efficiency and/or in a regulatory capacity may contribute to the development of a pathologic process that plays out over a span of several years [51].

We observed significantly elevated FI levels in our patients compared to the control group. FI is a soluble 88 kDa protein, responsible for the cleavage of C4b and C3b. FI activity depends on cofactors such as C4BP, CFH, CR1, and MCP. In addition, a polymorphism near the *FI* gene has recently been associated with risk of advanced AMD [62] and elevated expression of FI under the influence of inflammatory cytokines such as interleukin-6 (IL-6), IL-1, and TNF-α has been reported [63].

The cleavage of C3b is a central step in all three activation pathways of the complement system. There is some evidence that a polymorphism in the C3 (Arg^120^Gly) gene increases the risk of developing AMD along with the CFH Tyr^402^His polymorphism [64]. The presence of the C3 variant Arg^80^Gly was correlated to AMD [65]. Several complement proteins such as C1q, C3, C4, C5, CFH, membrane cofactor protein, decay accelerating factor, and fragments C3a and C5a are commonly found in drusen, AMD’s hallmark. Furthermore, recent data have suggested these deposits stimulate local activation of the complement system. This could lead to increased growth of deposits due to the strong chemotactic activity that results from the activation of certain fragments of the complement system (e.g., C5a and C3a) and a marked influx of inflammatory cells [66]. Higher plasma levels of Bb and C5a were observed in patients with advanced AMD compared to control individuals [51] confirming the continuous activation of the alternative pathway during AMD. The levels of these Bb and C5a fragments were not affected by different gene polymorphisms (*FH, FB, C2, C3, FD*, *FI*, and hypothetical gene LOC387715/age related maculopathy susceptibility-2 [*LOC387715/ARMS2*]) described as related to AMD risk. Amyloid substances present in the drusen of patients with AMD have the ability to bind to FI, probably inhibiting its activity in the complement regulatory cascade in the same way that these substances bind to CFH [67]. Therefore, FI dysfunction could accelerate C3 convertase generation, and the subsequent uncontrolled complement activation would lead to an intense local inflammatory response.

The initial response of the immune system is triggered by an infection or other offending agent and involves the release of molecules such as cytokines (IL-12, TNF-α, IL-1) and acute phase proteins [68,69]. CRP is an important acute phase protein in humans and has been under investigation because of its strong association with many inflammatory diseases. This plasma protein plays a role in innate immunity due to its ability to bind to microorganisms and activate the complement cascade. The plasma concentration of CRP usually does not exceed 0.1 mg/dL; however, this value can rapidly increase during inflammation, tissue injury, or extensive infectious processes, reaching 50 g/dl or more, due to its increased synthesis by the liver [70].

CRP activates the classical pathway through its interaction with C1q [71]. However, high levels of CRP (>150 mg/ml) efficiently inhibit the activation of complement [72]. CRP can also interact with CFH in a manner dependent on Ca^2+^ [73], and this binding enhances the regulatory ability of CFH in the alternative pathway, preventing the formation of C3 and C5 convertases [74-78]. In addition, CRP could form a complex with C4BP [79], acting as a FI cofactor, which in turn would degrade C4b and reduce the activation of complement by the degradation of C4b. Some studies [14,80-82] have correlated increased plasmatic concentrations of CRP with progression of AMD while others [21,83], including the present work, found no significant difference between the concentrations of CRP between patients with AMD and controls. The involvement of CRP in AMD disease development therefore remains a controversial issue.

Previous studies [81] have suggested that patients with AMD carrying the variant CFH His^402^ presented a higher CRP concentration locally in the eye than patients with the variant CFH Tyr^402^. Furthermore, CRP has been consistently found in drusen [79,81]. The CFH SCR 7 domain, where the CFH Tyr^402^His polymorphism is located, is also the binding site for CRP. The Tyr^402^His substitution could alter CFH’s ability to bind to CRP and other ligands and perhaps affect the level of local inflammation in the outer layers of the retina [39]. A defective interaction between CRP and complement factor H and factor H-like protein 1 is believed to intensify the inflammatory cascade [19]. Johnson et al. [81] have shown that individuals with the CFH His^402^ variant had higher levels of CRP in the choroid. Elevated plasmatic CRP levels and reduced serum CFH associated with obesity, hypertension, and smoking are considerable risk factors for AMD [43-45,84]. In 2008, Kim et al. [40] reported a correlation between plasma CRP and incidence of AMD, although none of the known CRP polymorphisms showed any correlation with the disease. These findings reinforce the significance of CRP as a marker of inflammatory disease processes but do not necessarily point to the participation of this protein in the etiopathogenicity of AMD. Local inflammation and immune-mediated events are critical to the development of drusen [42,84,85]. In our study, however, CRP levels remained normal in most individuals, and we observed no difference between the AMD and control groups. This leads us to conclude that we cannot systemically associate CRP levels with development of AMD.

In conclusion, we propose that the CFH Tyr^402^His polymorphism does not influence the plasma levels of CFH protein in patients with AMD or in the control group of normal individuals and that this polymorphism does not appear to be related to changes in the serum levels of other components of the alternative pathway of complement. However, patients and controls differ in the concentrations of FD (reduced in patients with AMD) and FI (increased in patients with AMD), which suggest some other factor participates in activating the complement system in this disease. Considering the i) high number of patients of this study and ii) the strong ethnic mix observed in the Brazilian population, differences observed in the plasma levels of crucial proteins for activating the alternative pathway found in patients with AMD may contribute to understanding the role of the complement system in the pathogenesis of this disease. As far as we know, this is the first study investigating this kind of association in a highly ethnically heterogeneous population.
